# Botulinum Neurotoxin A in the Treatment of Pharyngocutaneous Fistula after Salvage Surgery in Head and Neck Cancer Patients: Our Preliminary Results

**DOI:** 10.3390/curroncol29100557

**Published:** 2022-09-28

**Authors:** Maria Raffaella Marchese, Tiziana Di Cesare, Eugenio De Corso, Martina Petracca, Giuseppe Oliveto, Giovanni Almadori

**Affiliations:** 1 Unità Operativa Complessa di Otorinolaringoiatria, Dipartimento di Scienze dell’Invecchiamento, Neurologiche, Ortopediche e della Testa-Collo, Fondazione Policlinico Universitario A. Gemelli IRCCS, 00168 Rome, Italy; 2 Movement Disorders Unit, Fondazione Policlinico A. Gemelli IRCCS, 00168 Rome, Italy; 3 Sezione di Otorinolaringoiatria, Dipartimento Universitario Testa-Collo e Organi di Senso, Università Cattolica del Sacro Cuore, 00168 Rome, Italy

**Keywords:** botulinum neurotoxin A, pharyngocutaneous fistula, total laryngectomy, salvage surgery, pharyngolaryngectomy

## Abstract

*Objective*: To analyze the effect of intraparotid injection of botulinum neurotoxin A (BoNT-A) on salivary production and the course of pharyngocutaneous fistula (PCF) in post-radiation therapy salvage surgery. *Methods*: A total of 13 patients who had undergone total laryngectomy or pharyngolaryngectomy were treated with BoNT-A to both parotid glands, within three days from PCF onset. The salivary flow was evaluated using a subjective rating scale as the percentage of normal function from 0% (no saliva) to 100% (normal saliva flow), before injection, every day for 2 weeks, and once a week for three months. PCFs were monitored daily. *Results*: Spontaneous closure of PCF occurred in 7/13 (53.84%) cases 13.6 days (range: 7–18) after treatment; 6/13 (46.16%) patients needed revision surgery. Salivary flow significantly decreased in all patients seven days after injection (from 67.2% to 36.4%; *p* < 0.05). Patients who had undergone either conservative or surgical treatment did not differ in salivary flow before injection, whereas the mean percentages of salivary flow calculated at each time point after injection were different (*p* < 0.05). *Conclusions*: BoNT-A contributed to the closure of the fistula in most of our cases. The subjective perception of salivary flow predicted the closure of PCF. The mean time to closure may contribute to establishing the timing of PCF surgical treatment.

## 1. Introduction

Pharyngocutaneous fistula (PCF) is the most common complication of total laryngectomy or pharyngolaryngectomy (TFL) after prior concurrent cisplatin-based radiotherapy or cetuximab, or radiotherapy alone. The PCF consists of communication between the pharynx and the cervical skin around the surgical incision or, less frequently, the tracheal stoma. Breakdown of the mucosal suture can cause saliva to flow into surrounding soft tissues, delaying wound healing in such a saliva-soaked environment, which may induce hypoxia and chronic inflammation and frailty of the skin and vessels of the neck, leading to the formation of a fistula [1]. According to the most recent meta-analysis published in the international literature [2,3,4], the incidence of PCF is 14.3% for up-front total laryngectomy (TL), increasing to 27.6% for salvage TL or TFL, whereas it varies only from 8% to 22% in case of neotube reconstruction with a locoregional flap or microvascular free flaps. Numerous risk factors have been identified: previous radiochemotherapy or bioradiotherapy, pre-operative tracheotomy, bilateral neck dissection, postoperative sepsis, advanced stage of tumor, and systemic factors (i.e., sarcopenia, anemia, and hypoalbuminemia) are strongly related to the development of the fistula [2,5]. In addition to the neotube closure that represents the primary objective, salvage surgery often requires a complex reconstruction of the surgical defect with a large area of skin loss surrounding the tracheal stoma, tracheal stoma reconstruction, with or without distal trachea suspension, and the resurfacing of the inferior neck [6]. Different myocutaneous regional flaps (pectoral), fasciocutaneous propeller perforator flaps (IMAP), and microvascular free flaps, as it has been demonstrated, prevent postoperative fistulas, lowering the incidence to about half of the cases in total laryngectomy after radiotherapy [6,7,8]. Nevertheless, even if PCF occurrence is a high-impact complication leading to increased morbidity, delay in initiating oral diet, prolonged hospitalization, and increased costs of treatment [9,10], there are no shared guidelines for its treatment. Certainly, in the cases of PCF without major wound breakdown or vessel exposure, the common practice is to start with a conservative treatment because, as it is generally agreed, most PCFs respond well to this approach (56–90% successful rate), especially in non-irradiated patients. The conservative management traditionally consists of antibiotics and anti-inflammatory drugs, suspension of oral feeding with the position of a nasogastric tube or parenteral nutrition, and daily local wound care, including drainage of fluids from the fistulous tract, local cleaning with antibiotic solutions, chemical cauterization with a silver nitrate stick, removal of all necrotic tissue followed by curettage of the fistulous borders, and a pressure dressing above the neck flap [3,11]. Other conservative measures are negative-pressure wound therapy and hyperbaric oxygen therapy, even if their effectiveness, accessibility, and costs are not favorable. When conservative measures fail and the fistula persists, a surgical closure is indicated [12].

The role of botulinum neurotoxin A (BoNT-A) in salivary control by local injections into the major salivary glands of patients affected by neurological diseases (i.e., Amyotrophic Lateral Sclerosis, Parkison’s disease) or ENT movement disorders is widely accepted. In terms of the latter, post-traumatic sialocele [13] and idiopathic hypersalivation [14] have been described. In this regard, the beneficial effect of BoNT-A in the treatment of post-operative saliva-related complications has been previously reported [15].

The aim of this work was to specifically analyze the effect of BoNT-A on salivary production and its impact on the course of pharyngocutaneous fistula in salvage TL or total PL due to the failure of prior radiotherapy alone or with concurrent cisplatin or cetuximab.

## 2. Materials and Methods

Patients who had undergone salvage total laryngectomy or laryngopharyngectomy and affected by pharyngocutaneous fistula were recruited at the Otorhinolaryngology Unit of the A. Gemelli IRCCS University Hospital Foundation in Rome from January 2012 to January 2020. The exclusion criteria were: age <18 years, previous tracheostomy or neck dissection, neurological disorders, diabetes mellitus, immunosuppression state, or severe anemia. The drug was provided for off-label use by the hospital pharmacy upon submission of extensive documentation requested illustrating the advantages and the risks related to the therapy in each case. Written informed consent was obtained from all patients enrolled in the study. The presence of a fistula was established when salivary leakage through the neck or drainage was found. Moreover, an active test for fistula was performed with the administration of methylene blue dye orally on the fifth to seventh postoperative day, and any leakage through the neck or drainage was recorded as a fistula. According to the classification of Hawkes and Stell [16] we distinguished 3 types of fistula: fistula less than 0.5 cm in diameter (type 1), greater than 5 mm but less than 20 mm (type 2), and larger than 20 mm (type 3). At the appearance of the fistula the first treatment was conservative, consisting of antibiotics and anti-inflammatory drugs; suspension of oral feeding with the position of a nasogastric tube or parenteral nutrition, daily local medication, including drainage of fluids from the fistulous tract, removal of necrotic tissue, local cleaning with antibiotic solutions, chemical cauterization with a silver nitrate stick, and a compressive dressing. Moreover, within three days of the PCF onset, we injected the parotid glands with botulinum neurotoxin A (BoNT-A). We used onabotulinum toxin A (onabotA, Botox^®®^). The drug was delivered in vials of 100 mouse units (MU). Our protocol of therapy foresaw a total dose of 80 MU (40 MUI for each parotid gland divided into two points of injection). The injection was performed without anesthesia, under ultrasound guidance. After disinfection of the skin, a sterile ultrasound gel was applied to the skin. The first phase consisted of the precise location of the gland to be injected with ultrasound (high-frequency linear probe > 7.5 MHz). Then, a 1 mL syringe with a 29-gauge needle was introduced perpendicularly to the ultrasound transducer in-plane and a second injection, about 1–2 cm away from the first, was performed in the same gland. The effect on the salivary flow was evaluated using a subjective rating scale as the percentage of normal function—0% denotes no saliva and 100% denotes normal salivary flow—before injection, every day for 2 weeks, and then weekly for three months.

PCF was daily monitored by an objective examination of the fistula, considering its size and possible closure. Besides the clinical drooling examination and investigation of possible complaints (including side effects), a complete assessment of the head and neck area was performed. Tongue movements, eyes, lip and mouth closure, and swallowing were monitored weekly. The follow-up period lasted 12 weeks.

Statistical analysis was performed using commercially available software (Excel; Microsoft Corp, Redmond, Washington, DC, USA). Continuously distributed outcomes were summarized as the mean. Quantitative data were compared using Wilcoxon signed-rank and *t*-test. The significance level was set at 0.05.

## 3. Results

Based on the inclusion criteria, 13/14 (88.8%) subjects were eligible. A total of 2/13 (15.38%) were females and 11/13 (84.61%) were males, and the mean age was 70.8 years (range 66–78 years). A total of 12/13 (92.3%) had undergone total laryngectomy, and 1/13 (7.7%) total pharyngolaryngectomy and reconstruction with rotation flap (Table 1).

The mean number of days between surgery and PCF onset was 11.83 (range: 6–18 days). All cases were classified as PCF type 2. In all cases, the BoNT-A injection was performed a mean of 2.3 days after the onset of the PCF (range: 0–3 days). In 7/13 (53.84%) cases, the PCF was dry after a mean of 7 days (range: 4–10 days), and closure was noted a mean of 13.6 days after BoNT-A treatment (range: 7–18 days). In 6/13 (46.16%) subjects, we detected only a reduction in PCF size; therefore, surgical closure was required. We performed a direct suture of the pharyngeal mucosa defect in 1/6 (16.7%) cases, thanks to the fistula’s size reduction, and the internal mammary artery perforator (IMAP) propeller flap reconstruction in the remaining 5/6 (83.3%) patients. The mean time between the PCF occurrence and the surgical treatment was 27.8 days (range: 25–32 days). No patient experienced a worsening of the local condition of the fistula or an increase in its size.

**Figure 1:**
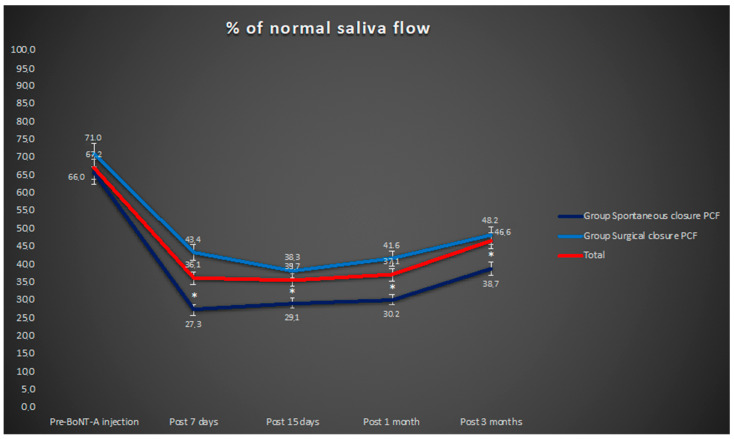
The mean percentage of normal saliva flow observed in all cases, in the group of subjects who demonstrated a spontaneous closure of PCF and in the group of subjects treated surgically; * *p* < 0.05 group PCF spontaneous closure vs. group PCF surgical closure.

Before BoNT-A injection, the mean percentage of normal salivary flow in all cases was 67.2% (range 50–75%). Seven days later the percentage decreased significantly (67.2% vs. 36.4%; *p* < 0.05). The scores measured 7 days, 15 days, one month, and three months after the treatment are shown in Figure 1. Before BoNT-A injection, the mean score of salivary flow obtained in the patients who had received only the conservative therapy of the PCF did not statistically differ from the one measured in the subjects who had surgery (66% vs. 71%). On the other hand, the mean percentages of normal salivary flow calculated at each time point after BoNT-A injection were statistically different between the two groups, with a decrease in the saliva flow significantly greater in the cases that showed spontaneous resolution of PCF (*p* < 0.05) (Figure 1). No severe side effects related to the treatment, such as dry mouth or dysphagia, were observed. Moreover, no patient presented dysphagia due to fibrous stenosis after either conservative or surgical treatment.

## 4. Discussion

We demonstrated that the injection of botulinum neurotoxin A in the parotid glands of head and neck cancer patients affected by salivary fistulas after salvage surgery decreased the subjective perception of the saliva flow. Moreover, the decrease in the saliva flow was significantly greater in the cases of patients who showed spontaneous resolution of the PCF. Finally, the mean closure time of the PCF in the same subjects was 14 days after the BoNT-A injection.

It is generally agreed that most salivary fistulas respond well to conservative treatment [17,18,19]. If the non-surgical measures are unsuccessful in sealing off the pharynx from the skin within three weeks, surgical closure should be considered. Overall, consistent with the current opinion, our usual first management of PCF is conservative. The latter, as mentioned above, traditionally consists of drugs, enteral feeding, and daily medication. It has been demonstrated [11] that this approach contributed to spontaneous closure of the PCF after upfront surgery. More exactly, it was observed that the small fistulas, without previous irradiation, responded well to conservative management, whereas large PCFs, mainly related to previous radiotherapy, do not close spontaneously with a conservative approach nor with primary closure. In this study we treated subjects who had undergone salvage surgery for laryngeal cancer complicated with a mid-size PCF, adding the chemical denervation of parotid glands with BoNT-A to the usual conservative approach. Recent studies [20,21] suggest that effective control of saliva flow was obtained with a total dosage of up to 100 MU BoNT-A into both glands. In keeping with these findings, in our cases, 80 MU of onabotulinum toxin A injected using the above-mentioned technique has been effective and devoid of side effects or damage to structures within the parotid region. Moreover, in agreement with the literature [20,22], an initial effect was already present after 7 days, reaching its full extent approximately 15 days later.

In the literature, the mean time of spontaneous closure of PCF after upfront surgery (without employing the BoNT-A) is 20 days, with a minimum of 5 days and a maximum of 28 days [11,23]. On the other hand, irradiated patients who had undergone salvage surgery showed only a decrease in diameter approximately after 4 weeks (wait-and-see period) [11,23]. Our results suggest that the association of intraparotid injection of BoNT-A to the traditional conservative treatment was effective in 54% of the subjects, all previously radio-treated, with a mean closure time of 14 days (minimum 5 days and maximum 18 days). The BoNT-A has already demonstrated its efficacy as an adjuvant in the treatment of PCF after upfront surgery in head and neck cancer patients. In this regard, previous research has reported that the healing period was greatly reduced by BoNT-A injection: in fact, approximately 4 days after treatment the fistula was dry and within 7 days it was closed [15].

The association of prior radiotherapy with the formation of a PCF in head and neck surgical patients has been well established [24]. Furthermore, despite a few authors [11] having demonstrated that pre-surgery irradiation represents a certain risk factor for PCF occurrence, it seems that it also constitutes an adverse condition for primary and secondary PCF closure because of its effects on tissue. Radiation is known to be toxic to normal tissues and impair surgical wound healing, which is significantly manifested microscopically by obliterative endarteritis and fibrosis. Radiotherapy affects the microvasculature of tissue, leading to subintimal fibrosis, endarteritis, and thrombus formation. This leads to impaired vascularity and increased fibrosis that diminish tissue oxygenation and alter wound healing [6,25].

The responses obtained in our group of irradiated subjects provided the finding that the reduction in the saliva flow induced by botulinum neurotoxin A in most cases contributes to the spontaneous closure of the PCF. Nevertheless, the effect on saliva flow was not uniform among the patients. Anyway, in the cases with a lower reduction in salivary flow, the intraparotid BoNT-A injection did not avoid, except in one case, a major second surgery using internal mammary artery perforator propeller flap for fistula closure and skin neck reconstruction.

Several authors [26,27] consider that there is no reason to wait any longer if PCF closure is not obtained in one month with medical management because the presence of a PCF prolongs the patient’s stay in the hospital and results in swallowing difficulty due to fibrosis in most cases. Moreover, the surgical dissection in the neck is much easier and the resulting defects in the pharynx and cervical esophagus are generally smaller [28]. Therefore, the length of conservative treatment represents a crucial point in PCF management. It is known that independently of the length of the conservative approach, its effectiveness is significantly different between irradiated and non-irradiated patients. The closure rate in non-irradiated patients is reported between 80–95%, while in irradiated patients it is between 44 and 82% [29]. Moreover Higashino et al. [30], regardless of the type of PCF treatment, reported a median closure time significantly longer (106 days) in irradiated cases compared to that in non-irradiated cases (65 days).

The maximum time that we recorded from the occurrence to the closure of the PCF was 18 days. These data are interesting because in irradiated patients this may represent the time limit within which to expect the spontaneous closure of the PCF and consequently to foresee the final treatment. Nevertheless, the persistence of a PCF beyond the maximum closure time does not mean that surgery should be expected at this point. In fact, when the adjuvant BoNT-A injection fails to repair a PCF, it may still reduce the size and promote effective surgical interventions.

Despite its strengths, the limitations of this study are the small number of cases, the comparison based on our previous case series and data from the literature because of the lack of a control group. Another important limitation of our study is that we considered patients’ subjective perception of salivary flow, which should be objectified in the future evaluation of the effect of botulinum toxin in head and neck cancer patients. Moreover, further research is necessary to compare the effect of BoNT-A on different sizes of PCF. In conclusion, data on efficacy acquired in this study can assist with the design of a future randomized clinical trial.

## Figures and Tables

**Table 1:** Demograhic, oncological, and treatment data of the cases.

Cases	Age	Sex	Primary Site	Primary Stage	Primary Therapy	Recurrent Therapy	Time to Closure (Days)	PCF Therapy
1	75	M	Supralgottic	IV	CT + RT	TL	15	BoNT-A
2	78	M	Glottic	IV	CT + RT	TL	7	BoNT-A
3	68	M	Glottic	IV	CT + RT	TL	12	BoNT-A
4	72	F	Transglottic	IV	CT + RT	TL	18	BoNT-A
5	73	M	Glottic	IV	CT + RT	TL	14	BoNT-A
6	58	M	Supraglottic	IV	CT + RT	TL	16	BoNT-A
7	66	M	Supraglottic	IV	CT + RT	TL	13	BoNT-A
8	69	F	Glottic	IV	CT + RT	TL	26	IMAP
9	70	M	Transglottic	IV	CT + RT	TL	27	IMAP
10	71	M	Supraglottic	III	CT + RT	PL	28	IMAP
11	69	M	Supraglottic	IV	CT + RT	TL	29	IMAP
12	74	M	Glottic	IV	CT + RT	TL	32	IMAP
13	67	M	Glottic	IV	CT + RT	TL	25	DS

CT = Chemotherapy; RT = Radiotherapy; TL = Total Laryngectomy; PL = Pharyngolaryngectomy; IMAP = Internal Mammary Artery Perforator (IMAP) propeller flap; DS = Direct Suture.

## Data Availability

The data presented in this study are available on request from the corresponding author. The data are not publicly available due to privacy reasons.
